# A Scoping Review of the Associations of Golf with Eye Injuries in Adults and Children

**DOI:** 10.1155/2016/7216325

**Published:** 2016-07-18

**Authors:** Evan Jenkins, Roger Hawkes, Andrew Murray

**Affiliations:** ^1^Sport and Exercise, the University of Edinburgh, St. Leonard's Land, Edinburgh EH8 8AQ, UK; ^2^European Tour Performance Institute, Virginia Water GU25 4LX, UK

## Abstract

*Introduction*. Sport presents a risk of ocular trauma and accounts for a significant number of eye injuries that require hospital admission. The sport of golf presents a risk to eyesight from fast moving objects such as golf clubs and balls. This study aims to investigate the associations of golf with eye injuries and the reasons that these injuries occur.* Material/Methods*. A literature search was conducted using the databases MEDLINE, Web of Science, SPORTDiscus, and PsycINFO. Grey literature was searched using the WHO international clinical trials registry platform, Google Scholar, and ProQuest. Data was extracted using a standardised form and summarised into a report.* Results and Discussion*. Twenty-three studies were found relating to eye injuries in golf. Injuries appear to be rare, but more frequent in men and children. Injuries resulted in high rates of enucleation and visual impairment. Children sustained more injury from golf clubs whereas adults sustained more injuries from golf balls.* Conclusion*. Efforts are needed to encourage golf participants to understand the risks of ocular and indeed other head injuries. Initiatives to provide appropriate supervision and education on this topic are merited. Further research is needed to investigate the circumstances of eye injury in golf and assess the effects of interventions aimed at reducing risk of injury.

## 1. Introduction

Ocular injury is a recognised risk of taking part in sporting activity. Sport is estimated to be responsible for between 25 and 40% of all ocular injuries requiring hospital admission [1, 2]. Golf presents a risk to eye health from the swinging of golf clubs and golf balls in flight, both of which can transmit considerable force [3]. However, to our knowledge no review assessing ocular injuries in the sport of golf has been undertaken.

Golf is played by over 20 million participants in Europe alone and has seen a large increase in facilities worldwide [4]. Despite this, golf has one of the lowest rates of reported musculoskeletal injury of sports players [5], but eye and head injuries have been reported in the literature [6].

This review aimed to collate available evidence regarding ocular injuries in golf and assess the associations of golf with ocular injuries and any patterns in the occurrence of such incidents. This may alert golfers to possible risks, assist stakeholders attempting to improve health and safety for golf players, and highlight any gaps in the existing literature.

## 2. Materials and Methods

### 2.1. Databases

The following databases and search platforms were used to search for studies as part of a wider scoping review: MEDLINE, PsycINFO, SPORTDiscus, Web of Science, Google Scholar, WHO international clinical trials registry platform, and ProQuest.

### 2.2. Eligibility Criteria

The following inclusion and exclusion criteria were utilised:

Inclusion criteria are as follows.

- Research articles not limited by geographical location, language, or setting.
- All age groups and both sexes of participants.
- Research that looks at the general population, as well as specific population groups.
- All forms of golf (including but not limited to 18 holes, 9 holes, driving range, and spectating).
- Research articles discussing ocular/eye injuries in the context of golf.
- Sources of information including primary research studies, reviews (including but not limited to systematic reviews, scoping reviews, and meta-analyses), guidelines, and grey literature to include unpublished and on-going trials, annual reports, dissertations, and conference proceedings.

Explicit exclusion criteria identified are as follows.

- Opinion pieces/opinions, magazine and newspaper articles, case reports, and papers with no data.

### 2.3. Search Strategy

Using the keywords “golf” AND “health”, a limited initial search for review articles was conducted using Google Advanced Search and SPORTDiscus. Fifty-six articles were retrieved from this search and were further analysed by the research team.

The team elected to maximise inclusivity by identifying “golf” as the primary search term. This term alone was used for the predominantly health related databases such as MEDLINE and PsycINFO. Secondary search terms were also included for Web of Science, Google Scholar, and SPORTDiscus, using the search terms “golf” AND “health OR illness OR injur^*∗*^ OR fitness OR mortality OR morbidity.”

For grey literature searches, the term “golf” was used for the WHO international clinical trials registry platform. The search terms “golf AND health OR illness OR injur^*∗*^ OR fitness OR mortality OR morbidity” were also used when searching ProQuest database, the World Golf Foundation, the Royal and Ancient, the British Journal of Sports Medicine, the American College of Sports Medicine, and the Faculty of Sports and Exercise Medicine websites using the Google Advanced function. Grey literature studies were included if keywords appeared in the title.

All articles identified with keywords in the title were reviewed. This search strategy allowed the inclusion of articles relating to all aspects of golf and health. The results of databases and search platforms initially found 4041 articles, which were reduced to 3167 once duplicate articles were removed.

Following this, relevant articles were sought through the process outlined below:

1. One reviewer (Andrew Murray) assessed all titles and abstracts applying the eligibility criteria. A second reviewer (Evan Jenkins) assessed 10% of these titles and abstracts, with excellent agreement. Following this, the full text of each remaining article was sourced and read, applying the same criteria by two reviewers (Andrew Murray and Evan Jenkins). Further articles were then identified from reading the reference lists of all relevant articles.
2. Tables that are similar to those recommended for scoping reviews by the Joanna Briggs Institute [7] were used for data extraction of all remaining articles (see Table 1). Authors were contacted directly when further specific data was needed.
3. Evidence extracted was collated and data synthesized relating to the number of cases, gender of participants, numbers needing surgery, and any serious visual impairment sustained.

## 3. Results and Discussion 

### 3.1. Study Selection

The scoping review search yielded 3167 studies. Three hundred and fifty-six studies were read in full with 23 found to be directly relevant to eye injuries in golf.

### 3.2. Study Characteristics

Twenty-three studies were included in this review, after applying the eligibility criteria. These comprised 7 retrospective studies [6, 8–13], 2 prospective studies [14, 15], 2 patient case series [16, 17], 1 review of sports-related eye injuries [18], and ten [19–28] studies which described ocular injuries that resulted from exposure to the contents of liquid filled golf balls. These 10 studies included 4 histopathology investigations [19–22], 2 patient case series [23, 24], 3 case notes [25–27], and 1 commentary report [28]. Finally, a commentary review by Brunette [29] explored the link between golf and health.

### 3.3. Results

Data from all retrospective studies [6, 8–13], both prospective studies by Jones and Gregory [14, 15], and the review of sports-related eye injuries by Portis et al. [18] were deemed most relevant to current golf participants. These studies report 102 patients who presented to accident and emergency departments with golf-related ocular injuries, during the period of 1990–2007.

Using a large cohort of 309 patients, Fradkin et al. [6] demonstrated that ocular injuries were the second most common type of injury sustained from golf-related trauma, accounting for 8.1% of all injuries. Furthermore, Gregory [14] demonstrated that golf-related ocular injury (GROI) accounted for 2.2% of all sports-related ocular injuries that were admitted to the accident and emergency department of Sussex Hospital. This was similar to results from the study by Jones [15], where GROI accounted for 2.4% of all sports-related ocular injuries. After viewing records of golf-related ocular injuries at a pathology laboratory, Portis et al. [18] report that 14.0% of sports-related ocular injuries were from playing golf. However, each of these studies only presented data on the total number of GROI admitted to tertiary health centres. Therefore, these papers were not used for further analysis as none can contribute to results in other categories.

Ten studies [19–28] described injuries occurring from the expansion of liquid filled golf balls, which are no longer in popular use. Therefore, these studies are not relevant to modern golf participants. The review by Brunette [29] only made mention of an incidental case of GROI in a commentary. These 10 studies were not included in the synthesis of results. Data by Millar [16] and Takatama et al. [17] are described separately as these case series cannot provide data on the incidence of injury.

In summary, 71 patients are reported to have suffered GROI. Fifty (70.4%) incidents occurred in males, 20 (28.2%) in females, and1 (1.4%) case of a 1-year-old where gender was not given (see Table 2). Twenty-one (29.6%) injuries were sustained from being hit by golf clubs and 50 (70.4%) from golf balls. Golf ball related trauma equated to 91.5% (43 cases) of all 47 adult injuries. Twenty-four (33.8%) injuries occurred in children of which 17 (70.8%) injuries were from being hit by golf clubs (children being defined here as aged 18 or under). Six injuries occurred in golf spectators, accounting for 8.5% of all cases (golf spectators were defined here as anyone not playing or practising golf).

As outlined in Table 3, the consequences of these injuries were often severe, with 55 (77.5%) injuries requiring surgery and 24 (33.8%) of these cases resulting in enucleation or evisceration of the eye. Blindness, in at least 1 eye, was reported in 27 (38.0%) patients of which 19 (70.4%) injuries were caused by impact from a golf ball and 8 (29.6%) from golf clubs. Furthermore, of the 6 studies which describe visual acuity, 34 (47.9%) out of the 71 patients had significant reductions in visual acuity below 20/40 Snellen, including those with no light perception [8–13]. As most studies reported visual acuity using the 20-foot US Snellen notation, this same notation was used for this review [8, 9, 11, 12]. For studies using the 6-meter UK based notation [10, 13], visual acuity changes below 6/12 were recorded and used to demonstrate impairment in visual acuity. This review regards any changes to visual acuity as a result of GROI as significant. Globe rupture was reported in 26 cases of which 22 (84.6%) were the result of impact from golf balls.Orbital fractures were reported in 5 studies [8, 9, 11–13] and were present in 30 (50%) out of the 60 patients reported.

When collating these results with those of Millar [16] and Takatama et al. [17], a total of 89 ocular injuries were reported, with 64 (71.9%) cases occurring in males. Impact from golf balls resulted in 60 (67.4%) ocular injuries and 1 additional case describes injury from a golf cart crash. Numbers requiring surgery drop to 69.7% (62 cases) whilst rates of eye blindness remain relatively even at 33 (37.1%) cases.

Ten studies [19–28] discuss incidences of ocular injury from old style golf balls exploding and how the liquid centres commonly cause ocular injury in children. The studies commonly describe an “opaqueness” and corneal fibrosis that occur after contact with, what was found to be, inorganic crystalline material. The most recent report of this type of injury was from 1976 by Lucas et al. [20].

### 3.4. Discussion

Ocular injuries in the sport of golf are infrequent events which are associated with high rates of blindness and risk to sight. The setting of the included studies may partly explain the severity of the reported injuries, as each study took place in accident and emergency departments of either district hospitals or specialist eye hospitals [6, 8–13]. Further studies appraising eye injuries reported to golf course staff or primary care/family doctors may show a decreased severity of injury on average.

GROI occurs most often in males which may reflect the higher participation of men in golf [30]. It was also found that children sustained a greater number of injuries than adults; however, this may not be a fair estimate as 1 of the 6 studies used to collate results only collected data on children [10].

Children sustain more injuries from golf clubs through incidents that commonly occur away from golf courses and without adult supervision [10, 13]. This pattern highlights the need for adult supervision of children who use golf equipment. Adults must take such opportunities to teach children the correct golf etiquette and safety protocols, such as checking behind them before swinging a golf club.

Golf equipment should be kept safely out of reach of children to avoid the high number of injuries that occur outside of golf courses [9]. Townley et al. [13] suggest that large media campaigns and advertisements may assist in warning all owners of golf equipment about the dangers these items pose to children and of the need for supervision.

In contrast, adult injuries usually occur whilst playing golf and are often the result of impact from a golf ball. This suggests that further enforcement of golf health and safety protocols must be made clear to anyone present on a golf course [10]. This may be achieved through safety briefings, signs or posters situated around the golf course, or campaigns as described above. Jayasundera et al. [10] note that player experience had little to do with the risk of receiving ocular injury and therefore recommendations to lower the risk of injury must be accessible to players of all levels.

These measures should also be extended to golf spectators as there is some incidence of eye injury in this population. Safety should be considered in the layout of driving ranges. For example, play should not be in close proximity to walkways and protective measures such as signage and netting should be implemented [12]. When play is likely to occur in close proximity to crowds, such as driving ranges, it is possible for spectators to be injured by a golfer's backswing. Any danger of this can be addressed by implementing policies and safety measures to minimise danger from swinging golf clubs. This could include safety lines which spectators must stand behind during play.

As discussed by Jayasundera et al. [10], education of appropriate safety protocols is essential to preventing injury. For example, golfers who hit a wayward ball are expected to shout “fore” to warn passersby. If not appropriately informed, many golfers then choose to look up to see the approaching ball and increase injury risk. Therefore, all golfers must be taught to look down and shield their head to avoid such injury.

Finally, protective eye equipment is discussed by most studies and ultimately would guarantee some level of protection [12]. As mentioned by Townley et al. [13], fast moving golf balls can be difficult to avoid and such equipment would aid anyone unable to shield themselves. Mandatory use of these may be difficult to impose; however, such equipment may have a role when teaching children or introducing newcomers to the sport [12].

### 3.5. Strengths and Limitations

This review is limited by a relatively small patient population and having only 2 studies [13, 17] making comments on the specific circumstances that resulted in ocular injury. As a result, it is difficult to make recommendations regarding health and safety in golf. Additionally, 3 studies only collect information from specific patient populations or of certain modes of injury [6, 9, 12]. Therefore, evidence used to make recommendations is weak due to the limited availability of data. Furthermore, as the studies discussed use data from accident and emergency and trauma departments, they likely underreport less significant injuries that were not admitted to these departments. Therefore, this may reduce the amount of data regarding minor eye injuries in golf.

However, all retrospective studies [6, 8–13] collect data over at least 5 years, making these the best available evidence regarding the incidence of traumatic ocular injury in golf. All recommendations must be made from the best available evidence and modified as new evidence emerges. This review is the most current collection of evidences regarding eye injuries in golf.

The papers collected for this review have been peer-reviewed by 2 independent reviewers with experience working as physicians in primary and secondary care settings and experience working with golfing populations.

### 3.6. Recommendations for Future Research

Further cross-sectional studies are needed both to identify the cause of injury and to provide any comment regarding the circumstances surrounding each incident. Where practical, studies to assess the incidence of eye injuries would provide a better understanding of the frequency with which GROI occur. This may be achieved through large cohort studies of golfers and recording of all GROI. However, to most effectively quantify GROI, prospective studies taking place in well-circumscribed geographic areas are needed. Such studies should take place in both emergency and ophthalmology departments.

The overall results derived by this review are dependent on the reporting and publication of GROI, regardless of whether the studies provide data of GROI incidence. However, underreporting of these injuries is very likely as many clinicians will choose not to report GROI through published case series, such as that done by Takatama et al. [17]. Therefore, future studies may wish to use appropriate ICD-10 codes to help gather further data of eye injuries through billing and payer records.

Research is needed to assess golf players' knowledge of health and safety rules. This may include assessing the knowledge of golf players at all levels, regarding their understanding of golf safety, and then comparing this to knowledge held by the general public. This information may highlight areas of golf safety that are poorly understood and therefore need to be taught. It may also demonstrate gaps in public knowledge and help guide the health and safety information issued with golf equipment. Efforts must then be made to adapt these messages for children.

Future reviews may also wish to focus on all head and face injuries sustained in golf. These injuries are likely to occur under similar circumstances to eye injuries but will encompass a larger patient cohort. Alternately, collecting data through family doctors or general practitioners may also include a larger number of patients but with less severe injuries.

## 4. Conclusion

There is little published data regarding the incidence of GROI. Though GROI occurs relatively rarely, the severity of injury is high, with many cases resulting in loss of vision or enucleation [2]. Children sustain most of their ocular injuries from golf clubs, whilst adults are most frequently injured from golf balls. Children should have constant adult supervision whilst using golf equipment and be taught rules regarding safety. Anyone buying golf equipment should gain an understanding of these rules to minimise risk of injury.

The use of protective eye equipment should be considered and may be appropriate when teaching children. Considering the large population of golfers, targeted media campaigns may best inform players of how to avoid serious trauma [9]. Future studies should focus attention on all head injuries in golf and decide which aspects of health and safety are most important to advertise to players and the general public.

## Figures and Tables

**Table 1:** Criteria used to extract data.

Author	Names of all authors involved in the study.
Article title	The full title of the study.
Year of publication	The year the study was published.
Type of publication	Which category of publication the study is in, for example, journal article, commentary, or letter.
Country	In which country was the study written?
Aims of the study	A description of questions the study is attempting to answer.
Population	How many people did the study discuss?
Methodology/methods	What methods did the study use to collect data?
Concept	What topic does the study investigate?
Context	In what setting does the study take place?
A priori themes: golf participation, physical activity and health, golf and physical activity, longevity, cardiorespiratory, metabolic, and musculoskeletal, accidents, illness, wellness, mental illness, special populations	Does the study discuss any of the outlined themes?
Any emergent themes not covered previously	Does the study note any points regarding themes not previously mentioned?

**Table 2:** Demographic information of patients who sustained golf-related ocular injuries.

Study	Number of cases	Patient group			Average age *(years)*
		Male	Female	Children *(<18 years)*	
Burnstine and Elner [8]	9	6	3	2	35.1
Fradkin et al.^⧫^ [6]	16	—	—	16	—
Gregory^⧫^ [14]	2	2	0	—	32.0
Hink et al. [9]	11	6	5	11	10.2
Jayasundera et al. [10]	11	8	3	4	26.3
Jones^⧫^ [15]	2	—	—	—	—
Mieler et al. [11]	8	6	2	2	44.4
Millar^⧫^ [16]	7	5	2	3	29.0
Park et al. [12]	22	16	6	0	48.5
Portis et al.^⧫^ [18]	11	—	—	—	—
Takatama et al.^⧫^ [17]	11	9	2	—	47.0
Townley et al.^*∗*^ [13]	10	8	1	4	29.0

*Total *	*120*	*66*	*23*	*42*	*33.5*

^⧫^Not included in result synthesis.

^*∗*^Case of a 1-year-old child where gender was not specified.

**Table 3:** Consequences of golf-related ocular injury.

Study	Cause of injury		Number needing surgery *(number of eyes)*	Number of globe ruptures *(number of eyes)*	Blindness in at least one eye *(number of eyes*)
	Golf club (number of children injured)	Golf ball (number of children injured)			
Burnstine and Elner [8]	2 (2)	7 (0)	9	8	6
Fradkin et al.^⧫^ [6]	—	—	—	—	—
Gregory^⧫^ [14]	0 (—)	2 (—)	—	—	—
Hink et al. [9]	10 (10)	1 (1)	9	1	2
Jayasundera et al. [10]	4 (4)	7 (0)	7	5	2
Jones^⧫^ [15]	—	—	—	—	—
Mieler et al. [11]	2 (0)	6 (2)	8	4	3
Millar^⧫+^ [16]	4 (1)	3 (1)	2	2	2
Park et al. [12]	0 (0)	22 (0)	15	5	7
Portis et al.^⧫^ [18]	8 (—)	3 (—)	11	—	11
Takatama et al.^*∗*⧫^ [17]	—	7 (—)	7	—	4
Townley et al. [13]	3 (1)	7 (3)	7	3	7

*Total*	*33 (18)*	*65 (7)*	*75*	*28*	*44*

^⧫^Not included in synthesis of results.

^+^Incidence of child striking glass with a golf club resulting in a perforating injury.

^*∗*^One incidence of ocular injury from a golf cart crash.
